# On the applicability of the Tubulin-Based Polymorphism (TBP) genotyping method: a comprehensive guide illustrated through the application on different genetic resources in the legume family

**DOI:** 10.1186/s13007-020-00627-z

**Published:** 2020-06-12

**Authors:** Luca Braglia, Floriana Gavazzi, Laura Morello, Silvia Gianì, Peter Nick, Diego Breviario

**Affiliations:** 1 CNR- National Research Council, Institute of Agricultural Biology and Biotechnology-IBBA, Via Alfonso Corti 12, 20133 Milan, Italy; 2 Department of Molecular Cell Biology, Botanical Institute, Karlsruhe Institute of Technology (KIT), Fritz-Haber-Weg 4, 76131 Karlsruhe, Baden-Württemberg Germany

**Keywords:** Genotyping, Legumes, β-Tubulins, Intron Length Polymorphism, TBP (Tubulin Based-Polymorphism)

## Abstract

**Background:**

Plant discrimination is of relevance for taxonomic, evolutionary, breeding and nutritional studies. To this purpose, evidence is reported to demonstrate TBP (Tubulin-Based-Polymorphism) as a DNA-based method suitable for assessing plant diversity.

**Results:**

Exploiting one of the most valuable features of TBP, that is the convenient and immediate application of the assay to groups of individuals that may belong to different taxa, we show that the TBP method can successfully discriminate different agricultural species and their crop wild relatives within the Papilionoideae subfamily. Detection of intraspecific variability is demonstrated by the genotyping of 27 different accessions of *Phaseolus vulgaris*.

**Conclusions:**

These data illustrate TBP as a useful and versatile tool for plant genotyping. Since its potential has not yet been fully appreciated by the scientific community, we carefully report all the experimental details of a successful TBP protocol, while describing different applications, so that the method can be replicated in other laboratories.

## Background

DNA-based plant identification performed at different taxonomical levels is now regularly supporting classical taxonomy based on morphological criteria and the availability of voucher specimens as certified material of reference. Several DNA-based methods have been developed, and more will likely follow in the near future, especially driven by the massive advances in genomics and next-generation sequencing. Even so, precise taxonomic recognition will remain an open challenge in plants, because of their more permissive interspecific barriers, leading to a smaller taxon gap as compared to animals [1]. In fact, outcrossing resulting from pollen dispersal may lead to events of interspecific hybridization that, in combination with allopolyploidy, can lead to the emergence of new species, sometimes favored by an improved adaptation to ongoing environmental changes. Ideally, molecular markers capable of tracking the process of speciation would need to be developed. Routinely, the emergence of a new species, when successfully established, is recorded in a more conventional way based on morphological or physiological features and the whole process is summarized in a phenogram. Time elapsing is indeed the dynamic variable that distinguishes phylogenesis from taxonomical classification. To this regard, it is increasingly evident that plastid genome sequences, abundantly used for DNA barcoding studies to define phylogenic relationships, must be accompanied by the use of nuclear DNA sequences [2]. In particular, when the evolutionary history of a hybrid or an allopolyploid species must be recorded, it is now clear that, although used in several multiple combinations, plastid markers do not suffice and the use of other sequences is required [3–5]. Ribosomal Internal Transcribed Spacer (rITS) has been largely reconsidered in several groups of important plants such as herbals and ornamentals [6–8]. ITS-based species recognition primarily relies on the amplification of nuclear rDNA spacers, carried out by the use of primers designed on conserved flanking sequences. A similar combined approach, based on the exploitation of highly repetitive regions present in both nuclear and organellar DNA, is nowadays carried out by genome skimming [9, 10]. However, plant species is just one of the taxonomical ranks of importance for classification. Discrimination of subspecies, hybrids, varieties and ecotypes is likewise important [11, 12]. In addition, while several molecular markers have been developed for cultivated plants, the research on markers useful for the characterization of orphan crops (traditional crops that have fallen out of use), as well as of crop wild-related species, is still dragging [13]. In crops, current molecular techniques are mainly applied to the genetic identification of species and varieties. Simple Sequence Repeats (SSR) and Single Nucleotide Polymorphisms (SNPs) markers are the two mostly used genotyping techniques for assessing difference at variety level. Neither is used for classification at species level, where, on the opposite, DNA barcoding based on either plastid genes or nuclear ribosomal sequences, is largely applied. Additional techniques suitable for exploring both intra- and inter-species variations are: High Resolution Melting (HRM) analysis [14], sequence-related amplified polymorphism (SRAP) markers [15], or conserved DNA-derived polymorphism (CDDP) markers [16]. HMR is particularly successful when genotyping is carried out by multiple SNPs, or other assays based on epigenetic variations but it suffers from the prerequisite of knowing a priori the DNA target sequence. On the other hand, SRAP and CDDP show several advantages such as highly resolved and scorable banding patterns, obtained by standard PCR reactions which require no further laboratory treatment, no need for nucleotide sequencing of the molecular markers, a number of amplicons that correlates quite well with the ploidy level and high interspecies transferability. Their major drawback is the failure of detecting variation in highly inbred species, sometime counterbalanced by their success in the recognition of hybrids origin. Based on length polymorphism, these kind of markers can take advantage from the random distribution in the genome of members of a gene family [16].

**Fig. 1:**
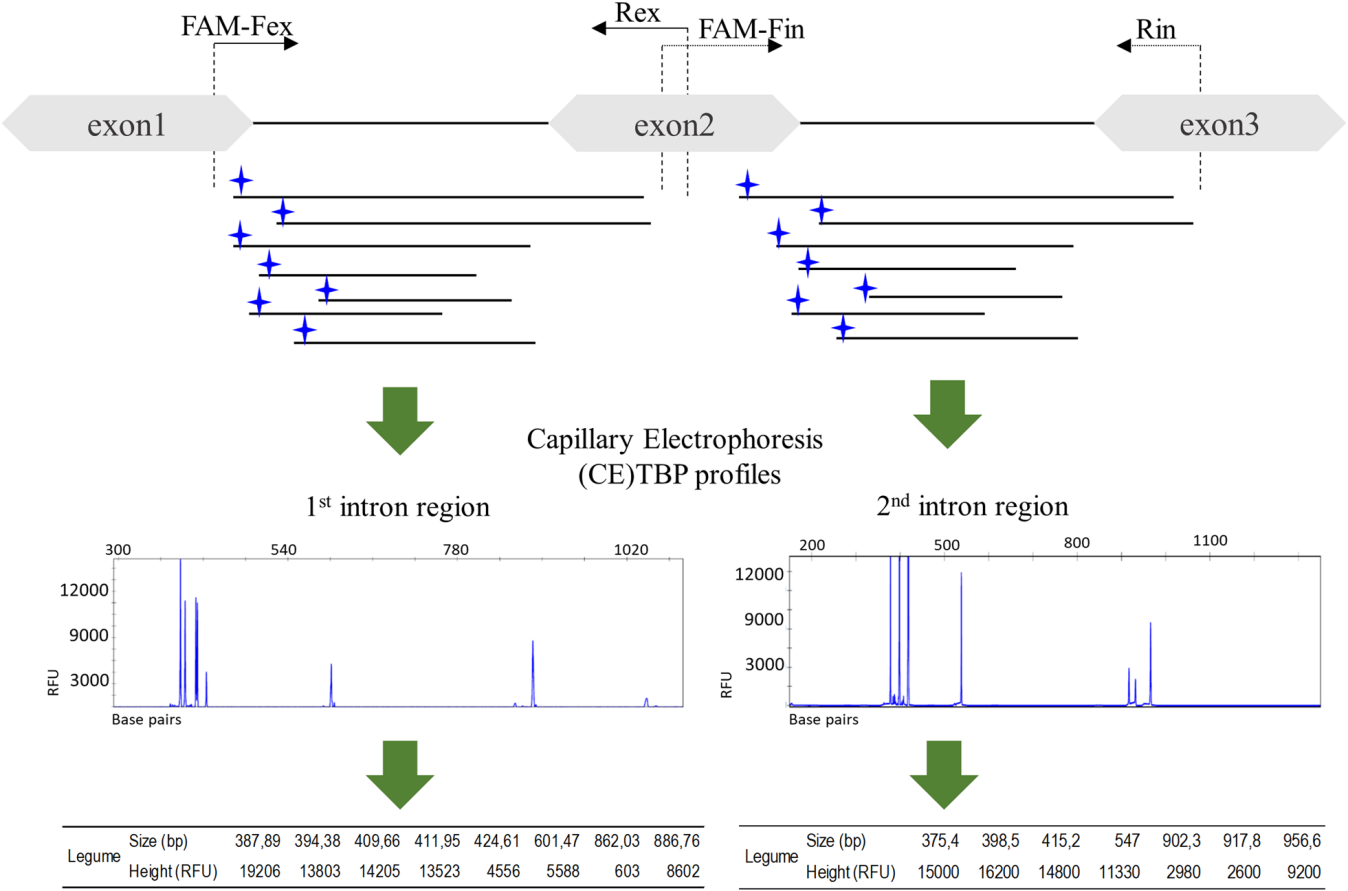
The TBP fundamentals and technique: a conceptual graph

Molecular ecology in plants is another field where suitable marker techniques are required to answer ecological questions. Here, the lack of interest, resulting from the scarce economic relevance of under-investigated ecological niches, is often associated to the absence of available genomic or genetic information, essential to design suitable molecular methods. This calls for the development of widely and arbitrarily applicable DNA markers, capable of deciphering genomic identities by simple molecular reactions with no need for a priori sequence information and a posteriori DNA nucleotide sequencing. Introns, located at conserved positions in the coding sequences of different members of the same gene family, can offer a convenient solution. They may vary in length (ILP for Intron Length Polymorphism), as well as nucleotide composition. ILP markers have been developed from various gene regions in many plant species. Largely applied to gene tagging, diversity analysis, genes association and comparative studies, they rely on variations in the untranslated portion of the genome [16–19]. Tubulin-Based Polymorphism (TBP) is an ILP marker that exploits the variation in the intron length of the different members of the beta-tubulin gene family. As shown in Fig. 1, a couple of primers capable of annealing to the conservative beta-tubulin exon boundary sequences can trigger, in a typical PCR reaction, the amplification of the intervening intron sequences, thus leading to the production of a set of amplicons, variable in length and number, depending on the analysed species or variety. Therefore, the same primer pair can successfully profile the genome of any plant of interest. Since plants typically contain two beta-tubulin introns, the power of discrimination can double. Recently, a TBP method applicable to vertebrates using a primer pair different from that used in plants has also been developed [20]. Application on fungi is instead hindered by the low number (often only one) of beta-tubulin genes and a far less conserved genomic organisation. Successful applications of TBP have been demonstrated in fields as diverse as genomic profiling within diverse botanical groups, such as grasses (*Eleusine indica*, *Phalaris arundinacea*, *Poa trivialis*, etc.), ornamentals (*Rosa* spp) and trees [21, 22]; parental identification, hybrid origin recognition and genetic relationships reconstruction [23]; ploidy level characterization [24]; qualitative and quantitative determinations of plant ingredients in feed [25]. Nevertheless, TBP as method for plant genotyping remains only occasionally utilized by the scientific community [26–28]. This may have been caused by a couple of reasons: an incorrect, oversimplified application of the protocol and the use of the original, less discriminating primers pair [29]. Hereby, we are thus providing all the experimental requirements and details to make the TBP method more readably accessible to laboratories involved in plant genotyping. To demonstrate its discriminative capacity, we have used the taxonomically challenging, or even recalcitrant, family of the Fabaceae as a case study.

The family Fabaceae or Leguminosae is the third-largest family of flowering plants, with a broad range of life forms extremely diverse in habits and cosmopolitan in distribution. It is divided in six subfamilies: Caesalpinioideae, Cercidoideae, Detarioideae, Dialioideae, Duparquetioideae, and Papilionoideae [30]. This latter is the largest one, comprising up to 14,000 species. Largely cultivated as crops and forages for their high protein content, legumes contribute significantly to total world food production. The genetic studies and the domestication history of legumes have played a vital contribution in the human life quality improvement. Their importance is partly responsible for the large number of available phylogenetic studies [30–34], essential for understanding the origin and diversification of this resource of economic and ecological relevance. In addition, the increased presence of legumes in ex situ and in situ germplasm collections, hosting unique landraces and wild relatives is crucial for future perspectives in both science and agriculture. In fact, the genomic fingerprinting of these wild or wild-related resources may assist in the development of breeding programs that could eventually lead to the production of more resilient crops, able to cope with the challenges of global climate change, or nutritionally improved, functional food, enriched for the presence of specific biomolecules. A central issue in the sustainable conservation of the plant genetic resources (PGR) is the knowledge of the genetic diversity present in gene bank collections and the subsequent exploitation of the genetic materials by breeding programs [35]. In fact, morpho-agronomical, biochemical and molecular analyses have been widely adopted in the germplasm characterization and species genetic diversity evaluation in legumes [36]. Despite this effort, only a small percentage of these collections have been characterized. Due to its flexibility of application at different taxonomical levels, TBP can represent a convenient tool for a first level classification. The purpose of this paper is thus to provide detailed experimental information for a vaster appreciation of the TBP method in the context of a potentially important application to Leguminosae.

## Methods

### Plant material

The plant material used in this study, listed in Table 1, was organised into three different experimental groups, to provide evidence for the capability of the TBP method of genotyping samples belonging to different taxonomic levels, within the papilionoids. The first experimental group included a core Crop Wild Relatives (CWR) seed collection (22 accessions), provided by the Botanical Garden of the Karlsruhe Institute of Technology (KIT) that have organised, by a national consortium of four botanical gardens, the gathering, cataloguing and ex situ conservation of CWR all over Germany. Accession numbers are reported in Table 1 according to the WEL (Wildpflanzen für Ernährung und Landwirtschaft) [37], the National Gene Bank for German Crop Wild Relative Species [38]. To maximise the number of species representing the large Papilionideae subfamily, additional legume crops, widely cultivated in Europe and currently available in our laboratory seed collection, were also included into this group (Table 1). For the CWR accessions the tribal classification of Smýkal et al. [34] was used.

**Table 1:** List of the plant material included in the present study and organised in three different experimental groups of analysis

Code	Latin name	Tribe/Section	WEL Ac. N°/Cultivar/Line/Ecotype	Form	ChN	N	Source origin-Breeding details^1^
*Group 1 Crop wild relatives*							
AVL	*Anthyllis vulneraria* L.	Loteae DC.	SW-5-404-2013	W	12	2	KT
ACC	*Astragalus cicer* L.	Galegeae (Bronn) Dumort	SW-3-57-2010	W	32	4	KT
AGY	*Astragalus glycyphyllos* L.	Galegeae	SW-5-446-2013	W	16	2	KT
CVR	*Coronilla varia* L.	Loteae	SW-3-483-2012	W	24	4	KT
CYS	*Cytisus scoparius* (L.) Link	Genisteae (Bronn) Dumort	SW-3-216-2011	W	48	4	KT
GTN	*Genista tinctoria* L.	Genisteae	SW-3-163-2010	W	96	4	KT
GLM	*Glycine max* L. Merr.	Phaseoleae (Bronn) DC.		C	40	2	IB
HGR	*Hedysarum grandiflorum* Pall.	Hedysareae DC.		C	16	2	IB
LLN	*Lathyrus linifolius* (Reichard) Bässler	Fabeae Rchb.	SW-6-141-2013	W	14	2	KT
LPR	*Lathyrus pratensis* L.	Fabeae	SW-3-408-2012	W	28	4	KT
LSL	*Lathyrus sylvestris*L	Fabeae	SW-5-300-2012	W	14	2	KT
LTB	*Lathyrus tuberosus*L	Fabeae	SW-3-429-2012	W	14	2	KT
LVR	*Lathyrus vernus* Bernh.	Fabeae	SW-6-138-2013	W	14	2	KT
LCC	*Lens culinaris* ssp. *microsperma* (Baumg.) NF.Mattos	Fabeae	‘Castelluccio’	C	14	2	IB
LCM	*Lens culinaris* ssp. *microsperma* (Baumg.) NF.Mattos	Fabeae		C	14	2	IB
LPE	*Lotus pedunculatus* Cav.	Loteae	SW-6-81-2012	W	12	2	KT
LAB	*Lupinus albus* L.	Genisteae		C	50	2	IB
MFA	*Medicago falcata* L*. x varia*	Trifolieae (Bronn) Endl.	SW-6-65-2012	W	16	2	KT
MLU	*Medicago lupulina* L.	Trifolieae	SW-1-114-2012	W	16	2	KT
MSA	*Medicago sativa*L.	Trifolieae		C	16	2	IB
MAL	*Melilotus albus* Medik.	Trifolieae	SW-3-363-2011	W	16	2	KT
MOF	*Melilotus offcinalis* (L.) Pall.	Trifolieae	SW-3-440-2012	W	16	2	KT
OVC	*Onobrychis vicifolia* Scop.	Hedysareae		C	28	4	IB
PSA	*Pisum sativum*L.	Fabeae		C	14	2	IB
PSF	*Pisum sativum* ssp. *arvense* (L.) Asch	Fabeae		C	14	2	IB
TAL	*Trifolium alpinum* L.	Trifolieae		W	16	2	IB
TAR	*Trifolium arvense* L.	Trifolieae	SW-3-31-2010	W	14	2	KT
TBA	*Trifolium badium* Schreb.	Trifolieae		C	14	2	IB
TCA	*Trifolium campestrev* Schreb.	Trifolieae	SW-1-157-2013	W	14	2	KT
TMO	*Trifolium montanum* L.	Trifolieae		W	16	2	IB
TPR	*Trifolium pratense* L.	Trifolieae	SW-1-73-2011	C	14	2	KT
TRE	*Trifolium repens* L.	Trifolieae	SW-3-251-2011	C	32	4	KT
TST	*Trifolium striatum* L.	Trifolieae		W	14	2	IB
VAN	*Vicia angustifolia* L.	Fabeae	SW-3-491-2013	W	12	2	KT
VEP	*Vicia sepium* L.	Fabeae	SW-5-118-2011	W	14	2	KT
VFB	*Vicia fava* L. ssp. *faba*	Fabeae		C	12	2	IB
VFE	*Vicia faba* L. ssp. *equina* St. Amans	Fabeae		C	12	2	IB
VSA	*Vicia sativa* ssp. *sativa*	Fabeae		C	12	2	IB
VFM	*Vicia faba* L. ssp. *minuta* (hort. ex Alef.) Mansf.	Fabeae		C	12	2	IB
*Group 2 Arachis*							
ARC	*Arachis archeri* Krapov. & W.C.Greg.	Aeschynomeneae Sec. Erectoides Krapov. & W.C.Greg	PI604844	W	20	2	VT
ARD38	*Arachis duranensis* Krapov. & W.C.Greg.	Aeschynomeneae Sec. Arachis Krapov. & W.C.Greg	PI10038	W	20	2	VT
ARD60	*Arachis duranensis*	Aeschynomeneae Sec. Arachis	PI30060	W	20	2	VT
ARD67	*Arachis duranensis*	Aeschynomeneae Sec. Arachis	PI30067	W	20	2	VT
ARD88	*Arachis duranensis*	Aeschynomeneae Sec. Arachis	K7988	W	20	2	VT
ARY39	*Arachis hypogaea* L.	Aeschynomeneae Sec. Arachis	PI109839	C	40	4	VT
ARY42	*Arachis hypogaea*	Aeschynomeneae Sec. Arachis	PI261942	C	40	4	VT
ARY60	*Arachis hypogaea*	Aeschynomeneae Sec. Arachis	PI119060	C	40	4	VT
ARY47	*Arachis hypogaea*	Aeschynomeneae Sec. Arachis	PI155247	C	40	4	VT
ARY90	*Arachis hypogaea*	Aeschynomeneae Sec. Arachis	PI262090	C	40	4	VT
ARYF	*Arachis hypogaea* ssp. *fastigiata*	Aeschynomeneae Sec. Arachis	Argontine	C	40	4	VT
ARYH	*Arachis hypogaea* ssp. *hypogaea*	Aeschynomeneae Sec. Arachis	NC6	C	40	4	VT
ARI	*Arachis ipaensis* Krapov. & W.C.Greg.	Aeschynomeneae Sec. Arachis	K30076	W	20	2	VT
ARM	*Arachis macedoi* Krapov. & W.C.Greg.	Aeschynomeneae Sec. Extranervosae Krapov. & W.C.Greg	GKP10127	W	20	2	VT
ARP	*Arachis paraguariensis* Chodat & Hassl.	Aeschynomeneae Sec. Erectoides	PI9640	W	20	2	VT
ARR	*Arachis rigonii* Krapov. & W.C.Greg.	Aeschynomeneae Sec. Procumbentes Krapov. & W.C.Greg	PI10097	W	20	2	VT
ART	*Arachis triseminata* Krapov. & W.C.Greg.	Aeschynomeneae Sec. Triseminatae Krapov. & W.C.Greg	GK12881	W	20	2	VT
*Gruop 3 Phaseolus*							
PlN	*Phaseolus lunatus* L.	Phaseoleae	Lima type - Clone L30	C	22	2	M
PvMH	*Phaseolus vulgaris* L.	Phaseoleae	‘Marozzo H’	C	22	2	M- Sarconi bean PGI Ecotype
PvMD	*Phaseolus vulgaris*	Phaseoleae	‘Marozzo D’	C	22	2	M- Sarconi bean PGI Ecotype
PvPB	*Phaseolus vulgaris*	Phaseoleae	‘Poverella B’	C	22	2	M- Sarconi bean PGI Ecotype
PvPG	*Phaseolus vulgaris*	Phaseoleae	‘Poverella G’	C	22	2	M- Sarconi bean PGI Ecotype
PvVF	*Phaseolus vulgaris*	Phaseoleae	‘Verdolino F’	C	22	2	M- Sarconi bean PGI Ecotype
PvVE	*Phaseolus vulgaris*	Phaseoleae	‘Verdolino E’	C	22	2	M- Sarconi bean PGI Ecotype
PvC	*Phaseolus vulgaris*	Phaseoleae	‘Ciuoto’	C	22	2	M- Sarconi bean PGI Ecotype
PvBB	*Phaseolus vulgaris*	Phaseoleae	‘Bianco di Bagnasco’	C	22	2	M- Cuneo bean PGI Ecotype
PvG	*Phaseolus vulgaris*	Phaseoleae	‘Giulia’	C	22	2	M- Italian commercial cultivar
PvP5	*Phaseolus vulgaris*	Phaseoleae	P500	C	22	2	M- BC3F7 (Giulia x(Giulia x G6388))
PvBN	*Phaseolus vulgaris*	Phaseoleae	‘Bonello’ Accession N 1511	C	22	2	M- Istituto ricerche orticole di Minoprio
PvHE	*Phaseolus vulgaris*	Phaseoleae	‘Heidi’	C	22	2	M- Heidi Bohn family, german bean grower
Pv39	*Phaseolus vulgaris*	Phaseoleae	L39	C	22	2	M- BCF4 (Heidi x (HeidixTaylor’s Horticultural))
Pv41	*Phaseolus vulgaris*	Phaseoleae	L41	C	22	2	M- BCF4 (Heidi x (HeidixTaylor’s Horticultural))
Pv42	*Phaseolus vulgaris*	Phaseoleae	L42	C	22	2	M- BCF4 (Heidi x (HeidixTaylor’s Horticultural))
PvT	*Phaseolus vulgaris*	Phaseoleae	‘Taylor’s Horticultural’	C	22	2	M- Taylor’s Dwarf Horticultural bean
PvGR	*Phaseolus vulgaris*	Phaseoleae	‘Greensleeves’	C	22	2	M- String, green bean commercial cultivar
PvEL	*Phaseolus vulgaris*	Phaseoleae	‘El Nũna 2001’	C	22	2	M- Peruvian popping bean
Pv58	*Phaseolus vulgaris*	Phaseoleae	SEL 358	C	22	2	C- G6616x(G4485xBATB32)
Pv82	*Phaseolus vulgaris*	Phaseoleae	SEL 582	C	22	2	C- DOR60xXAN112
Pv91	*Phaseolus vulgaris*	Phaseoleae	SEL 591	C	22	2	C- DOR60xXAN112
Pv22	*Phaseolus vulgaris*	Phaseoleae	SEL 922	C	22	2	C- G4090x(G4090x(G4090xRAB56))
Pv49	*Phaseolus vulgaris*	Phaseoleae	SEL 949	C	22	2	C- G4822x(G4822x(G4822xRAO17))
PvB8	*Phaseolus vulgaris*	Phaseoleae	BAT 881	C	22	2	C- (G3834xG2045)x(G3627xG5481)
PvL9	*Phaseolus vulgaris*	Phaseoleae	L905	C	22	2	C- BAT881 x(F2 (A55XG06388))
PvP	*Phaseolus vulgaris*	Phaseoleae	‘Pinto’	C	22	2	C- Pink bean cultivar
PvV	*Phaseolus vulgaris*	Phaseoleae	‘Viva’	C	22	2	C- SutterPink x(Red MexicanUI-351 x(RMUI-35xPI203958))

WEL Ac. N°: WEL Gene Bank accession number; Form, cultivated (C) or wild (W); ChN, Chromosome number; n, ploidy; PGI, Protected Geographic Indication

The taxonomic authority is reported at the first mention and according to the International Plant Names Index (IPNI- ipn.org)

^1^KT, Botanical Garden of the Karlsruhe Institute of Technology (KIT); IB, Institute of Agricultural Biology and Biotechnology (IBBA-CNR); VT, Department of Biological Sciences of the Virginia Tech University; M, Mediterranean Germplasm Database, Institute of Biosciences and Bioresources (MGD-IBBR-CNR); C, Centro Internacional de Agricultura Tropical (CIAT)

A second experimental group (Table 1) was defined by a single genus, here represented by 17 different *Arachis* accessions. Five different taxonomical sections of wild and cultivated species, subspecies and varieties, reported according to Bertioli et al. [39], were included. This plant material was provided in form of gDNA extracted from seed pools of each accession by the Department of Biological Sciences of the Virginia Tech University (Blacksburg, USA) and supplied according to their own Plant Introduction numbers (PI) [40, 41]. The cytological groups (genomes), section and ploidy level presented in the results and listed in Tables 1 and 3 are reported according to Stalker HT [42].

Finally, the application of the TBP method involved a third experimental group made up by 27 different bean (*Phaseolus vulgaris*) accessions, collected from different sources and including European local landraces as well as germplasm from the American gene pool, and one *Phaseolus lunatus* as outgroup. Seed material was provided by CIAT, Centro Internacional de Agricultura Tropical (Palmira, Colombia) [43] and by the MGD-IBBR, Mediterranean Germplasm Database of the Institute of Biosciences and Bioresources (Bari, Italy) of the Italian National Research Council (CNR). Table 1 reports the list of the bean germplasm inclusive of the available breeding details. Referring to the Sarconi bean PGI (Protected Geographic Indication) ecotypes, two distinct seed pools were considered for each accession (Marozzo H and D; Poverella B and G; Verdolino F and E).

### Tissue homogenization, DNA extraction and quality assessment

A pool of seeds for each analysed accession is ground to a fine powder (5–10 μm) applying 30 Hz for 1 min and 20 s by the TissueLyser II homogenizer (Qiagen, Germany) equipped with steel jars, including 1 stainless steel bead, 20 mm in diameter. One hundred and fifty milligrams of seed powder are subsequently sampled from each accession.

The DNA extraction is performed using a modification of the DNeasy Plant Mini Kit protocol (Qiagen, Valencia, CA, USA) as proposed by Braglia et al. [25] for the DNA extraction of complex matrices of plant origin. Briefly, the standard lysis buffer is replaced by a volume of 400 μL of guanidinium hydrochloride buffer (Buffer CF, NucleoSpin^®^ Food kit, Macherey–Nagel). Lysis is carried out at 65 °C for 30 min with continuous shaking, in the presence of 400 µg of RNase A and 20 µg of Proteinase K. The precipitation step is performed by adding 130 µl of P3 Buffer (DNeasy Plant Mini Kit, Qiagen, Valencia, CA, USA), and incubating the solution on ice for 10 min. The remaining purification procedures are carried on the robotic workstation Qiacube^®^ (Qiagen, Germany) according to product specifications for plant tissues, following the DNA Plant Mini standard procedure. DNA is eluted in 100 μl Tris–EDTA buffer and stored at − 20 °C until used.

The quality of the purified DNA is determined both by spectrophotometric UV absorbance estimation with Nanodrop 2000C (Thermo Fisher Scientific, Germany) and through 1% w/v agarose gel electrophoresis. The DNA concentration is estimated with the Qubit^®^ dsDNA BR Assay Kit on a Qubit 1.0 fluorometer (Thermo Fisher Scientific, Germany), following the manufacturer’s instructions.

While different kind of protocols are valid for the extraction of gDNA from seeds, the procedure just described was found to be more efficient in removing polysaccharides, polyphenols and other plant metabolites that could interfere with the subsequent PCR reaction.

For the *Arachis* group of samples, already provided as gDNA solution, the quality of the extracts was assessed as described above.

### TBP amplification protocol

As graphically summarized in Fig. 1 the TBP technique is based on PCR amplification of two different intron regions (1st and 2nd) of the plant β- tubulin genes. The two regions can either be analysed separately, setting up distinct reactions with the suitable couple of primers, or in combination, thus including the second exon flanked by the two introns. The current plant material was analysed for the 1st and the 2nd intron regions separately. Use of the combined version of the TBP technique (h-TBP) has been already reported by the authors with reference to the characterization of different *Camelina* species [44]. Table 2 shows the sequences of the primer pairs designed on the highly conserved, exon- intron boundary regions and the PCR amplification protocols developed according to the temperature of annealing of the primers.

**Table 2:** Primers sequences and amplification cycles for the TBP reaction

Intron region	Primer name	Primer sequence	Thermal profile
1st	Fex1	AAC TGG GCB AAR GGN CAY TAY AC	TBPTD1i
	Rex1	ACC ATR CAY TCR TCD GCR TTY TC	
2nd	Fin2	GAR AAY GCH GAY GAR TGY ATG	TBPTD2i
	Rin2	CRA AVC CBA CCA TGA ARA ART G	

T (°C)	Time		Cycle N°
*TBPTD1i*			
94	3 min		
94	30 s		
65	45 s	− 0.5 °C touchdown	× 14
72	2 min		
94	30 s		
57	45 s		× 15
72	2 min		
72	30 min		
*TBPTD2i*			
94	3 min		
94	30 s		
65	45 s	−0.7 °C touchdown	× 14
72	2 min		
94	30 s		
55	45 s		× 15
72	2 min		
72	30 min		

First and second intron of plant β-tubulin genes are amplified by the use of the Fex-Rex and Fin-Rin combination of primers, respectively. The forward primers, Fex and Fin, are labelled with a FAM fluorophore to allow resolution and analysis by capillary electrophoresis (CE). The CE-TBP profile is composed of different amplicons (peaks) each one characterized by a specific length, expressed in base pair (bp), and height, expressed in RFU. Typically, exon lengths are 396 (1st), 276 (2nd) and 668 (3rd) bp, for a total 1340 bp coding sequence.

The TBP reaction conditions are those reported earlier by Braglia et al. [25] for the amplification of total gDNA extracted from animal feeds. Here, we always refer to the analysis of TBP amplicons made suitable for resolution by capillary electrophoresis (CE-TBP) through the labelling of the forward primer of each pair in 5′ position with a FAM fluorophore (Thermo Fisher Scientific, Germany) [45].

In our experience, the range of gDNA amount used as template for the TBP reaction may vary from 5 to 200 ng, which is robust enough to cope with the wide variation in haploid genome size of land plants. In fact, this amount must be put in relation to the target copy number that also influences the necessary dilution rates that must be applied to the amplified products for the subsequent estimation of size and fluorescence intensity. In the present study, a range of 10 to 30 ng of total DNA was used as the template in a final reaction volume of 30 μl. The PCR reaction was performed in a Mastercycler (Eppendorf AG, Germany) by a touchdown thermal profile, specific for each of the two intron regions (Table 2), with a final concentration of 1 µM for each primer. Two aliquots of the same DNA extract were independently amplified for each analysed sample and control reactions without DNA template (negative control) were included in each experiment.

The potential presence of inhibitors of Taq polymerase activity, possibly co-purified during genomic DNA extraction, was assessed by the test described by Braglia et al. [25] The test allowed to identify, in the VWR Taq Polymerase Master Mix (2×, including 2 mM MgCl2) (VWR International), the lowest level of sensitivity to the inhibition effect, ensuring the absence of any interference in the TBP amplification reaction. The same inhibition test can be applied to evaluate other commercial polymerases, different from that used here. The test is based on a dose–response curve for amplification. Accordingly, increasing amounts (5−200 ng) of gDNA extracted from the plant material are added to the PCR amplification reaction for a 900 bp long DNA insert, hosted in a recombinant plasmid vector (100 pg), that can be conveniently amplified by a couple of universal primers. The amplicons are checked by agarose gel electrophoresis. If no inhibition effect is exerted by secondary compounds co-purified with the gDNA added to the plasmid insert amplification mix, the polymerase activity of the enzyme can be also considered valid for the TBP reaction.

### Capillary Electrophoresis

Four microliters of labelled TBP product (sample) are checked by 2% agarose gel electrophoresis, together with 0.2 µg of the GeneRuler™ 1 kb Plus DNA Ladder. In order to ensure that the amplicons amount fall into the detection range of capillary electrophoresis (CE), the suitable quantity of TBP product must be preliminary estimated and subsequently adjusted by appropriate sample dilution. A visual comparison performed between the fluorescent amplified TBP product and the DNA ladder, both loaded on the gel, indicates the proper dilution rate to be applied. For each sample, two different double-distilled-water dilutions, ranging from 2 to 50 folds, are prepared according to the visual inspection performed between the signal intensity of the DNA ladder and the sample. Two microliters of each diluted sample, and negative control, are then applied to the CE performed on a 3500 Genetic Analyser, (Thermo Fisher Scientific, Germany) after the addition of 0.18 µl of 1200 LIZ Size Standard (Thermo Fisher Scientific, Germany) and 17.82 µl of Hi-Di formamide (Thermo Fisher Scientific, Germany). Therefore, for each sample, two distinct CE runs are performed throughout.

We strongly recommend a denaturation step at 95 °C for 5 min in the presence of formamide, followed by a 3 min incubation on ice, in order to prevent the formation of secondary structures of the TBP amplicons that would otherwise alter fragments migration. The denaturation step in the presence of Hi-Di formamide preserves the single strand form of labelled DNA fragments, making them more suitable for CE analysis.

Amplicons are separated by CE according to the fragment analysis application of the instrument, through an 8-capillary-array of 50 cm (Thermo Fisher Scientific, Germany) filled with POP-7TM polymer (Thermo Fisher Scientific, Germany). The instrument was set as follows: dye set G5, oven pre-heat of 60 °C, injection and run voltage 10 kV and 8.5 kV respectively, injection time 3 s, and run time 5100 s.

### Data acquisition and analysis

Fluorescent TBP amplified fragments and the size-standard (1200 LIZ Size Standard, Thermo Fisher Scientific, Germany) migrate together across the polymer-filled capillary up to a detection window connected to a CCD camera, which detects and records the time-dependent fluorescence variations. The data collection and the primary analyses were performed using the 3500 Series Data Collection Software v. 3.1 (Thermo Fisher Scientific, Germany) by the use of an appropriate algorithm (Long Fragment 50_POP7) that evaluates the performance quality of the fragment analysis in terms of peaks resolution and sizing precision. A second specific software, the Gene Mapper Software v. 5.0 (Thermo Fisher Scientific, Germany), elaborates and processes the data, allowing the sizing and the release of a peak pherogram output. For each sample, the peak size in base pair (bp) is defined according to the fragment migration of the size-standard included in the run, and represents, in the pherogram, the peak position on the abscissa while the height of the peak represents the amount of fragment detected fluorescence (RFU). The peak area is also estimated by the software.

The analysis method of the Gene Mapper Software was set according to the “Advanced” parameters of the “Peak Detection Algorithm”. During sizing, the software performs a standard control comparing the size of the peaks of the standard included in the sample to those of the expected fragment sizes, listed in the size‐standard definition used for the analysis (1200 LIZ Size Standard). For each peak of the standard, the software also evaluates the relative height and the distance from the neighbours. Observed standard peaks that do not meet the expected pattern are discarded.

In the sample sizing step, the Gene Mapper Software assigns size values to the sample peaks (calling) by comparison with the size‐standard. The correct sizing of each sample peak is only allowed if the software verifies, across the sample pherogram, the correct assignment of both the preceding and the subsequent standard peaks, respectively. After sizing, the resulting CE-TBP pherogram is analysed according to a peak height threshold value defined as follows: only peaks with a height that exceed 50 RFU are recorded, while peaks exceeding 32,000 RFU are considered out of scale. CE-TBP runs exceeding the defined height threshold are excluded from the analysis.

Reliability and repeatability of the assay are always ensured by performing two independent TBP amplifications of the same gDNA extraction, and two different dilutions of each amplification, for each analysed sample. As a result, a single experimental sample is scrutinized by four different CE-TBP runs.

Three experimental groups of analysis (see the plant material section) were analysed and the resulting data for peak size and height, were collected by GeneMapper software and stored in a standard text data file for subsequent analysis. Samples were compared to each other within each analytical group, and the numerical data were organised in ascending order of magnitude according to the peak size. The peak size was considered as a marker and its presence/absence was scored as a binary matrix (1 for presence, 0 for absence) providing the input for the subsequent analytical steps. The PAST 3.26 (https://folk.uio.no/ohammer/past/) [46] an open source software was used to elaborate and integrate the data. The binary matrix was used to infer a β-component diversity estimation by the Whittaker’s pairwise comparison index [47] using PAST 3.26 software default parameters. The index provides a measure of the existing dissimilarity between and within species. The Shannon index was also estimated by the software, measuring the average degree of ‘‘uncertainty’’, within a group of samples, for predicting species assignment of randomly chosen individuals [48]. This uncertainty was defined as synonym for diversity [49] to evaluate the discrimination power of the TBP marker at different taxonomic ranks. A variance–covariance matrix was estimated to infer multivariate statistics and data plotting by Principle Component Analysis (PCA). The neighbor-joining algorithm [50] was used for the cluster analyses from the estimated dissimilarity matrices; the tree design was performed by Dendroscope software (version 3.5.10, Nov 2018, http://dendroscope.org) [51] and the statistical confidence of a particular group of accessions within the obtained trees was evaluated by bootstrap test with 1000 replicates (Additional file 1).

## Results and discussion

### Crop wild relatives

Unique and distinctive genomic profiles were obtained by applying the CE-TBP analysis to each of the species reported in the group 1 of Table 1, representative of 17 different genera of the large Papilionoideae subfamily. This allowed for rapid genotyping of forty-three different legume accessions (39 from the first experimental group plus sample ARY 39 from the second and samples PlN, PvP and Pv42 from the third group) that included wild germplasm (CWR) and cultivated crops, as well as local ecotypes with different chromosome numbers and ploidy levels (Additional file 2).

**Fig. 2:**
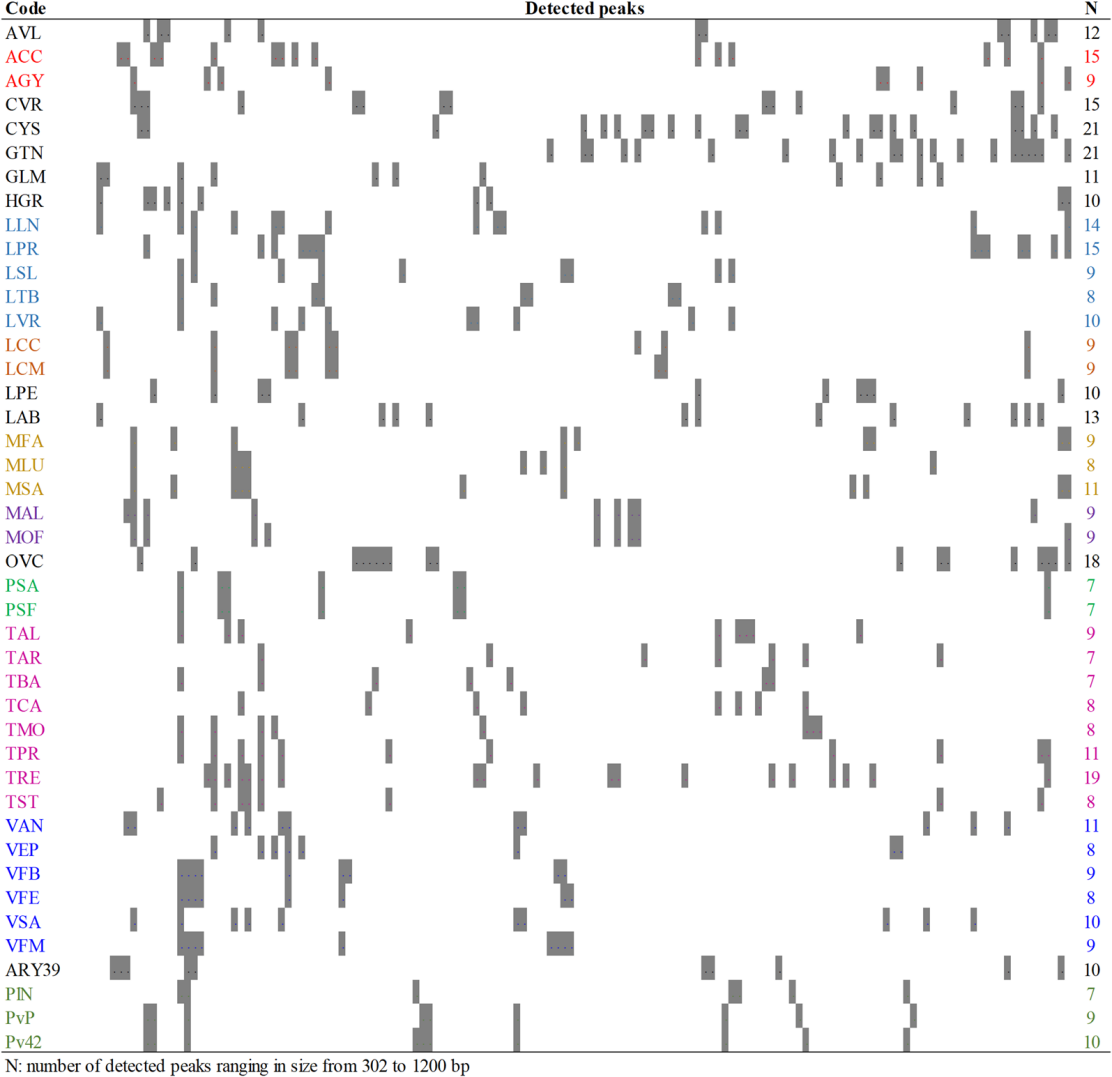
Diagram of the 1st intron CE-TBP profile of 43 papilionoid accessions

The diagram obtained from the alignment of the CE-TBP numerical profiles, organised in ascending order of peak sizes (Fig. 2, from left to right), provides a clear information about the length polymorphism present in the 1st intron of the β-tubulin genes, for all the analysed samples (Fig. 2). The peak-size estimation, ranging from 302 to 1200 bp, allows for a considerable discrimination power. A large variation in the number of detected amplicons, from 7 to 21, was recorded, that correlates with the ploidy status and the number of chromosomes (Table 1). In fact, a higher number of detected amplicons characterizes the tetraploid level of different species (Fig. 2). Accordingly, 21 peaks were detected in *Cytisus scoparius* and *Genista tinctoria,* tetraploid accessions with 48 and 96 chromosomes, respectively, while only 7 peaks were detected in some diploid species such as *Phaseolus lunatus, Trifolium arvense* and *T. badium*.

Shaded area corresponds to peaks. The accession name code is reported in Table 1. Same colour code refers to the same genus.

Overall, one hundred and thirty-nine polymorphic markers were detected (Fig. 2), reflecting the remarkable aptitude of the TBP method to score variability at both the inter-generic and the inter-specific level. All taxonomical ranks were distinctly genotyped comprising those accessions that were qualified as different subspecies or ecotypes (*Vicia faba* and *Lens culinaris* respectively). Only the two analysed subspecies of *Pisum sativum* (PSA and PSF) yielded identical TBP profiles. According to the diagrams of Fig. 2, species belonging to the same genera share some amplicons, while differing for others.

**Fig. 3:**
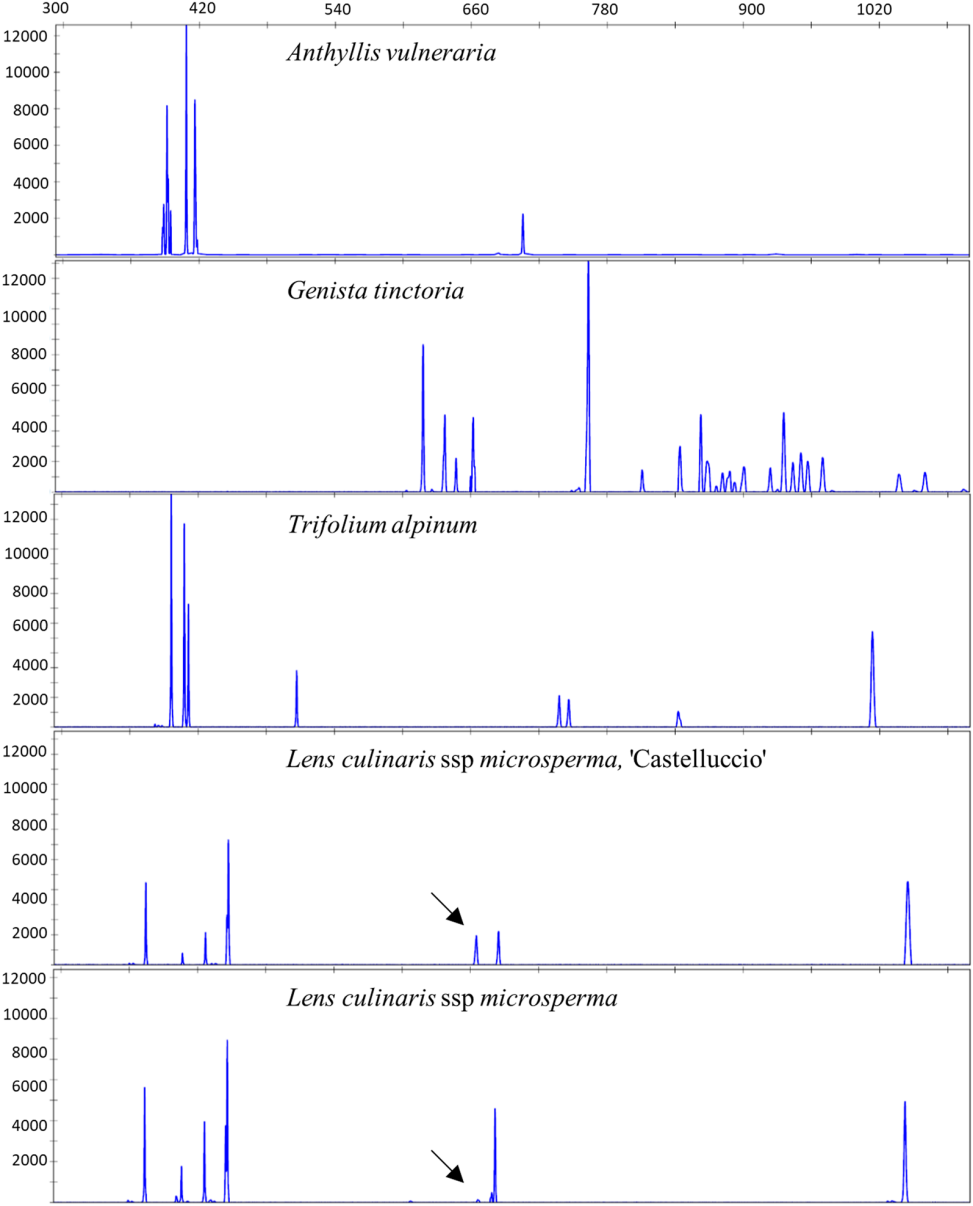
Five different representative accessions chosen to demonstrate the variation in CE-TBP peak profiles

The analysis of the first group of samples, defining the largest taxonomical category of this study, showed contrasting TBP patterns in terms of peak number, size and distribution, as exemplified for some representative samples in Fig. 3. Although the analysis of this group was limited to the 1st intron region, both relevant and minimal variations in the peak profiles could be easily appreciated.

The intensity of the recorded peaks is reported in RFU (Relative Fluorescence Units) on the ordinate and the size (in base pair) on the abscissa. Black arrows in the two bottom profiles point to the minimal differences that can be detected comparing the two ecotypes of *Lens culinaris*.

**Fig. 4:**
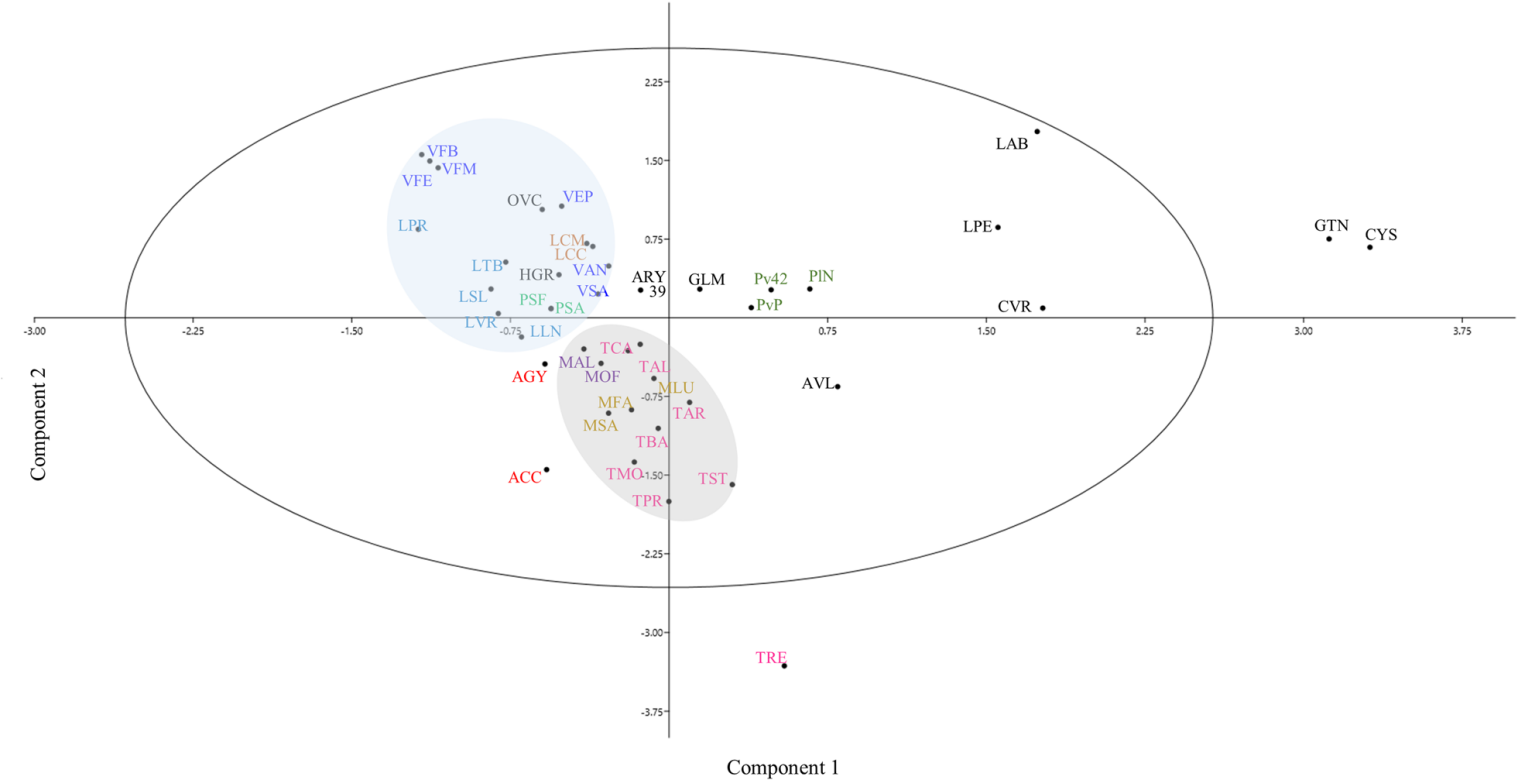
PCA bi-dimensional plot of 1st intron CE-TBP scored markers using a covariance matrix

Correlations among accessions were assessed by a Principle Component Analysis (PCA) (Fig. 4). The first principal component accounted for 29.30% and the second for the 28.87% of the total variance. Although moderate, these values assure a certain level of reliability to the dispersion shown, further substantiated by the finding that the overall distribution of the analysed accessions in the bi-plot reflects the taxonomic rank organization above genera (tribes), with few outliers. In fact, the 95% ellipse confidence interval isolates some distantly related accessions such as *G. tinctoria* (GTN) and *C. scoparius* (CYS*)*, both belonging to the Genisteae tribe, that cluster outside the ellipse, very likely because of their high number of β-tubulin introns (chromosome number reported in Table 1). Similarly, the widely cultivated lupine accession (LAB) belonging to the same tribe, clusters in the same plot quote although just inside the interval of confidence.

With reference to the whole sample distribution shown in Fig. 4, two main groups can be readily recognized. The first group (blue area in Fig. 4), enriched at species level, includes all the analysed accessions belonging to the genera *Lathyrus, Lens*, *Pisum* and *Vicia* within the tribe Fabeae. Despite the limited sizes of the taxon sampling, restricted to five species, this cluster corroborated the phylogenetic relationships proposed by Steele and Wojciechowski [52] and by Schaefer et al. [53], that places *Lathyrus*, *Lens* and *Pisum* as all nested in *Vicia.* The first group also comprises the two pea subspecies (PSA and PSF) that look alike, and the two lentil ecotypes belonging to the same subspecies (*Lens culinaris* ssp *microsperma*), that show a very close relation. The tribe Hedysareae is also included in this first group (HGR and OVC). The second group (grey area in Fig. 4) includes all the accessions belonging to the Trifolieae tribe. Within our analysed collection, *Trifolium* is the most represented genus with 8 different species. The contrasting ploidy level of the perennial tetraploid *T. repens* (TRE), strongly isolates this species from the others, placing it outside from the confidence ellipse. The remaining seven clovers are interspersed throughout the group together with the sweet clovers (*Melilotus* spp., MAL and MOF) and alfalfa accessions (*Medicago* spp. MLA, MFA and MSA).

The Phaseoleae tribe, including most of the domesticated grain legumes of worldwide importance like soybean and bean (GLM, PlN, PvP and Pv42) creates a moderately dense group of accessions. On the contrary, no clear clustering is visible for representatives of the remaining tribes, Aeschynomeneae (ARY39), Galegeae (AGY and ACC) and Loteae (AVL, CVR and LPE).

The 58% of the total variation of 43 papilionoids is plotted by the first two most informative PCs. The external ellipse includes the 95% of the confidence interval. Blue and the grey areas highlight species clustering within the tribes Vicieae and Trifolieae respectively. Same name colour code refers to the same genus.

The analysis of the genetic diversity present within our sampling was further complemented by the estimation of the Shannon index. This index increases as both the richness and the evenness of the analysed group increases, ranging from 1.61 to 2.94, values that assign a considerable discrimination power to the TBP method. Typical values found in most ecological studies are generally between 1.5 and 3.5, rarely greater than 4. As expected, the lowest values were found between accessions sharing identical TBP profiles (pea samples 1.61). These latter findings, likely attributable to the limited number of markers generated by the use of the 1st intron of the β-tubulin genes as the sole source of polymorphism, have encouraged and justified the addition of the 2nd intron in those assays that aim to address the extant diversity among samples of lower taxonomic ranks such as cultivars and ecotypes. Even though restrained to a low number of genera, compared to those currently attributable to the Papilionoideae subfamily, TBP was found highly effective in classifying unexplored genomes, allowing the inference of a primordial phylogenetic analysis in the absence of any preliminary sequence information. In this regard, referring to some *Lathyrus* species of the CWR material (LLN, LPR, LSL, LTB, LVR) the group distribution resulting from the TBP plot analysis supports the evidence of their closed genetic relationship to important legumes such as pea (PSA and PSF), as suggested by Schaefer et al. [53]. In fact, the TBP pattern of analysis showed the presence of some introns that are exclusively shared between *L. silvestris*, *L. tuberosus* and pea accessions (Fig. 2). Although they are incompatible for cross fertilization, Durieu and Ochatt [54] have already proposed the two grass pea species as potentially useful for somatic hybridization with pea, via protoplast fusion. The availability of shared molecular markers among the genera might be particularly appealing for pea breeding, in particular for those applications that aim to introduce some beneficial traits such as drought tolerance or perennial life [55].

### Peanuts (*Arachis* spp.)

The second group of analysis included 17 peanut accessions representing eight different species within a single genus. Subspecies, cultivars and breeding lines were also considered. Both intron regions were analysed by TBP and the scored peaks defined 79 polymorphic markers, 36 from the 1st intron region and 49 from the 2nd.

**Fig. 5:**
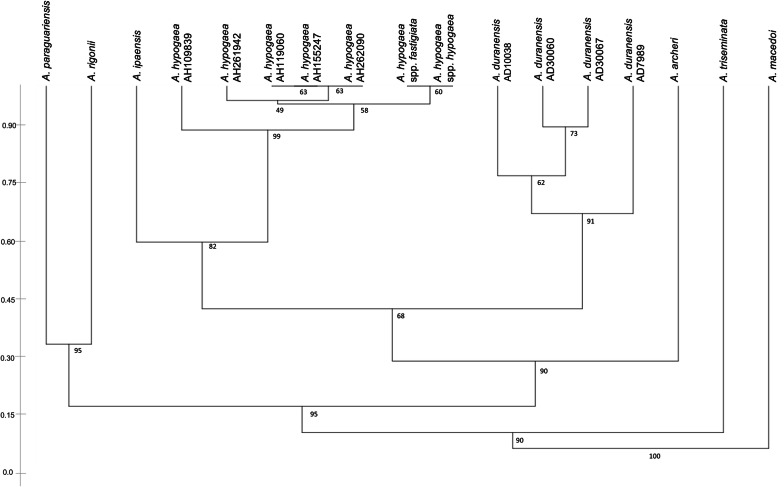
1st and 2nd CE-TBP neighbor-joining derived tree of 17 peanut accessions

The cophenetic correlation coefficient (r) calculated (p < 0.01) from the two, independently estimated, original distance matrices, resulted in a very good level of representation of the two intron regions (r = 0.92) thus supporting the complementarity of the information that is generated by intron size variations of the two diverse regions of the same gene. Therefore, the diversity measurements obtained by the combined analysis of the two introns, offered accurate and reliable results, supporting the use of the total number of markers for the cluster analysis distribution shown in Fig. 5.

Bootstrap values, with 1000 replicates, are reported at the branch nodes.

The phylogram in Fig. 5 groups the analysed samples according to the taxonomic sections distribution proposed by Bertioli et al. [39], based on morphology, cross-compatibility, geographic distribution and cytogenetics of the *Arachis*. In the tree, the section *Arachis* comprises the highest number of accessions (*A. ipaensis*, *A. duranensis* and *A. hypogaea*), including both annual and perennial species with different ploidy levels, that cluster together with the highest similarity values, within a main group that is clearly recognizable. Species that belong to sections Erectoides and Procumbentes (*A. archeri, A. paraguariensis* and *A. rigonii* respectively) are those most related to the main group whereas sections Extranervosae and Triseminatae, here represented by the *A. macedoi* and *A. triseminata* species, show the stronger genetic isolation within the genus (Fig. 5). The primordial phylogenetic association of these latter species to the cultivated peanut has been largely documented by others with the use of ITS lying between the 18S and 26S nuclear rDNA [56, 57], by the 5.8S rDNA sequences and using intron and microsatellite markers [58]. With reference to the main group, it is also shown that the TBP analysis is capable of distinguishing the allotetraploid cultivated accessions of *A. hypogaea,* and related subspecies, from the two wild diploids ancestors, *A. duranensis* and *A. ipaensis*, that, merged several thousands of years ago, contributed to their karyotype providing the A and B genomes, respectively (Table 3). In accordance, the highest number of TBP peaks (25–26) was detected within the accessions of *A. hypogaea,* whereas diploid accessions associates to a lower number of introns (13–21), as reported in Table 3.

**Table 3:** Total number of TBP markers obtained from the analysis of peanut accessions

Latin name	Number	n	Genome	Section	Form	TBP detected peak number^a^		
						1st intron	2nd intron	1st and 2nd
*A. archeri* Krapov. & W.C.Greg.	PI604844	2	E	Erectoides	W	10	11	21
*A. duranensis* Krapov. & W.C.Greg.	PI10038	2	A	Arachis	W	8	11	19
*A. duranensis*	PI30060	2	A	Arachis	W	8	9	17
*A. duranensis*	PI30067	2	A	Arachis	W	8	9	17
*A. duranensis*	K7988	2	A	Arachis	W	8	9	17
*A. hypogaea* L.	PI109839	4	AB	Arachis	C	10	15	25
*A. hypogaea*	PI261942	4	AB	Arachis	C	10	15	25
*A. hypogaea*	PI119060	4	AB	Arachis	C	11	15	26
*A. hypogaea*	PI155247	4	AB	Arachis	C	11	15	26
*A. hypogaea*	PI262090	4	AB	Arachis	C	11	15	26
*A. hypogaea* ssp. *fastigiata*	Argontine	4	AB	Arachis	C	11	14	25
*A. hypogaea* ssp. *hypogaea*	NC6	4	AB	Arachis	C	11	14	25
*A. ipaensis* Krapov. & W.C.Greg.	K30076	2	B	Arachis	W	6	9	15
*A. macedoi* Krapov. & W.C.Greg.	GKP10127	2	EX	Extranervosae	W	11	10	21
*A. paraguariensis* Chodat & Hassl.	PI9640	2	E	Erectoides	W	8	9	17
*A. rigonii* Krapov. & W.C.Greg.	PI10097	2	PR	Procumbentes	W	5	8	13
*A. triseminata* Krapov. & W.C.Greg.	GK12881	2	T	Triseminatae	W	6	10	16

n: ploidy, ^a^TBP peak number refers either to the two introns, individually analysed, or to their sum

Form, cultivated (C) or wild (W)

The cluster distribution shown in Fig. 5 also displays a moderate level of intra-specific variability within the analysed *A. duranensis* accessions and very low dissimilarity values recorded within *A. hypogaea*, where two samples corresponding to different subspecies appear indistinguishable. The intra-specific diversity has been also evaluated by the calculation of the Whittaker’s index, largely adopted in the evaluation of species richness of ecological communities, through the pairwise comparison of the presence/absence matrix of the TBP patterns. The index ranged from 0 to 0.263 when estimated among the four different cultivars of *A. duranensis* and among the seven accessions of *A. hypogaea*. Next, values were pooled together defining the overall range of intra-specific divergence. Conversely, the estimation of the general inter-specific variation scored much higher values, ranging from 0.238 to 1, when comparing all the eight species analysed within the same genus.

**Fig. 6:**
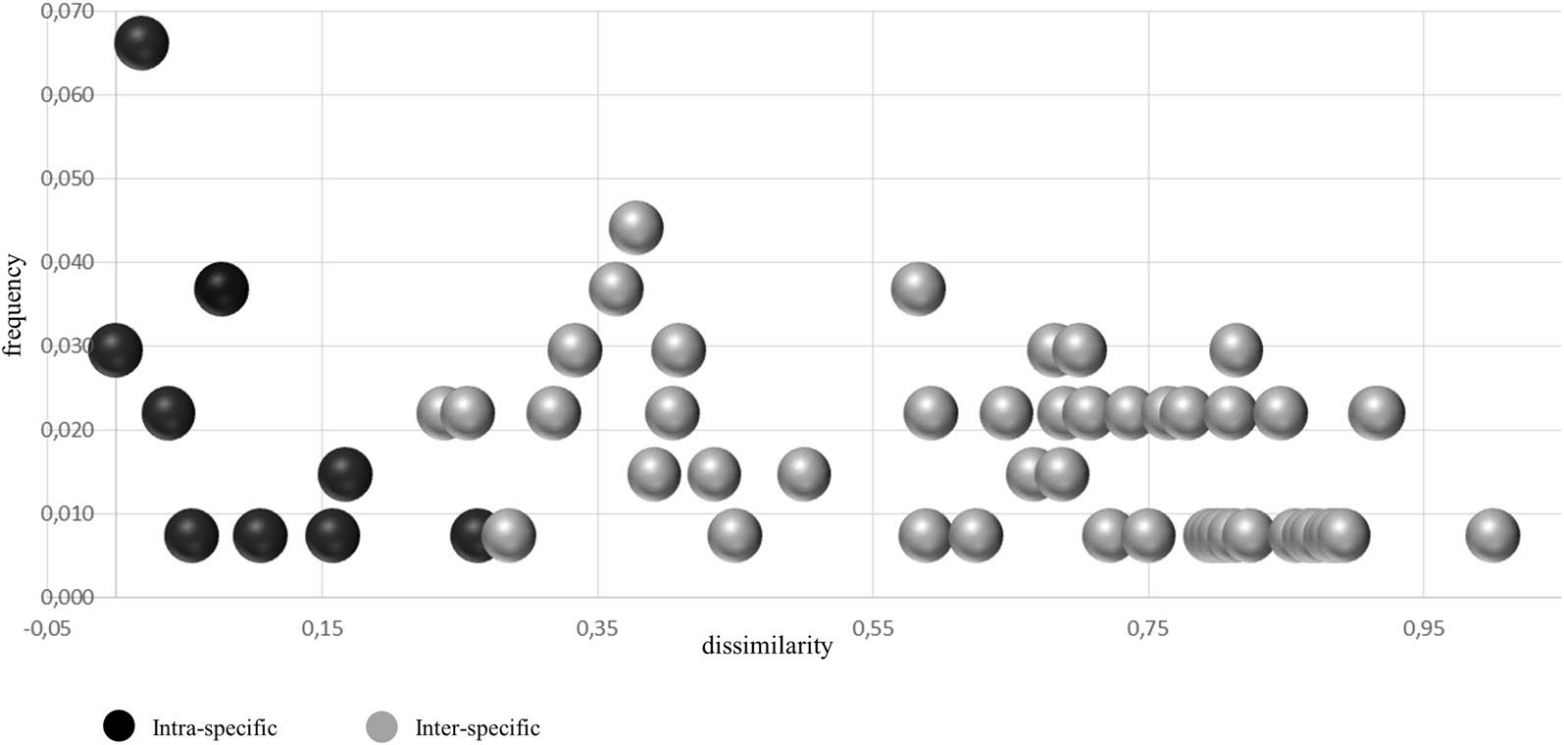
Frequency distributions constructed from the 1st and 2nd TBP regions among *Arachis* samples. The Whittaker’s diversity index estimated within (Intra) and among (Inter) species is represented by black and grey dots respectively

The frequency distributions of the estimated diversity index (dissimilarity) were used to evaluate the general overlap between intra- and interspecific variation. Figure 6 highlights the presence of a very limited overlap between the distributions for the intra- and the inter-specific dissimilarity, corresponding to a very restricted number of observations (1.5–2.2%). With reference to the strict definition of a barcoding gap, as applied to both DNA- barcoding and metabarcoding methods [59–61], the minimal overlap area found between the two intra- and inter-species distributions of Fig. 6, relates to the coalescent depths existing among the analysed species and cannot be interpreted as a limit of the TBP system for the reliable identification of species. In fact, according to different studies and to various methods, the identification success is particularly critical when working with closely related species [62, 63]. Furthermore, the success of DNA barcoding is expected to vary among groups depending on their evolutionary history, hybridization and polyploidization [64, 65]. If applied to *Arachis*, that includes long time cultivated and tetraploid accessions, TBP-based genome profiling always assures identification at species level, although additional, distinctive DNA profiles may also characterize subspecies (*A. hypogaea* ssp. *fastigiata* and ssp. *hypogaea*) and breeding lines (*A. hypogaea* PI109839 and PI261942).

### Beans (*Phaseolus vulgaris* L.)

A third group of analysis was defined to assay the intra-specific discrimination capacity of the TBP method when applied to lower taxonomic ranks such as cultivars and ecotypes. Two *Phaseolus* L. species were analysed: *P. vulgaris*, represented by 27 cultivars, commercial lines and landraces, and a single entry of *P. lunatus*, the Lima bean. Both introns of the β-tubulin genes were used for TBP profiling and peaks scoring identified 30 markers, 94% polymorphic and discriminant for all the analysed accessions, with few exceptions. The Italian landraces were genotyped by the TBP approach and despite the common evolutionary history and the narrow geographic distribution area of some gene pool (Sarconi bean PGI Ecotype, traditionally grown in Basilicata region), the method was capable of identifying specific DNA polymorphisms. Two distinct seed pools for each of the local ecotypes ‘Poverella’, ‘Verdolino’ and ‘Marozzo’ were also analysed showing full matching profiles for the first two ecotypes, and two allele variations in the third (data not shown).

**Fig. 7:**
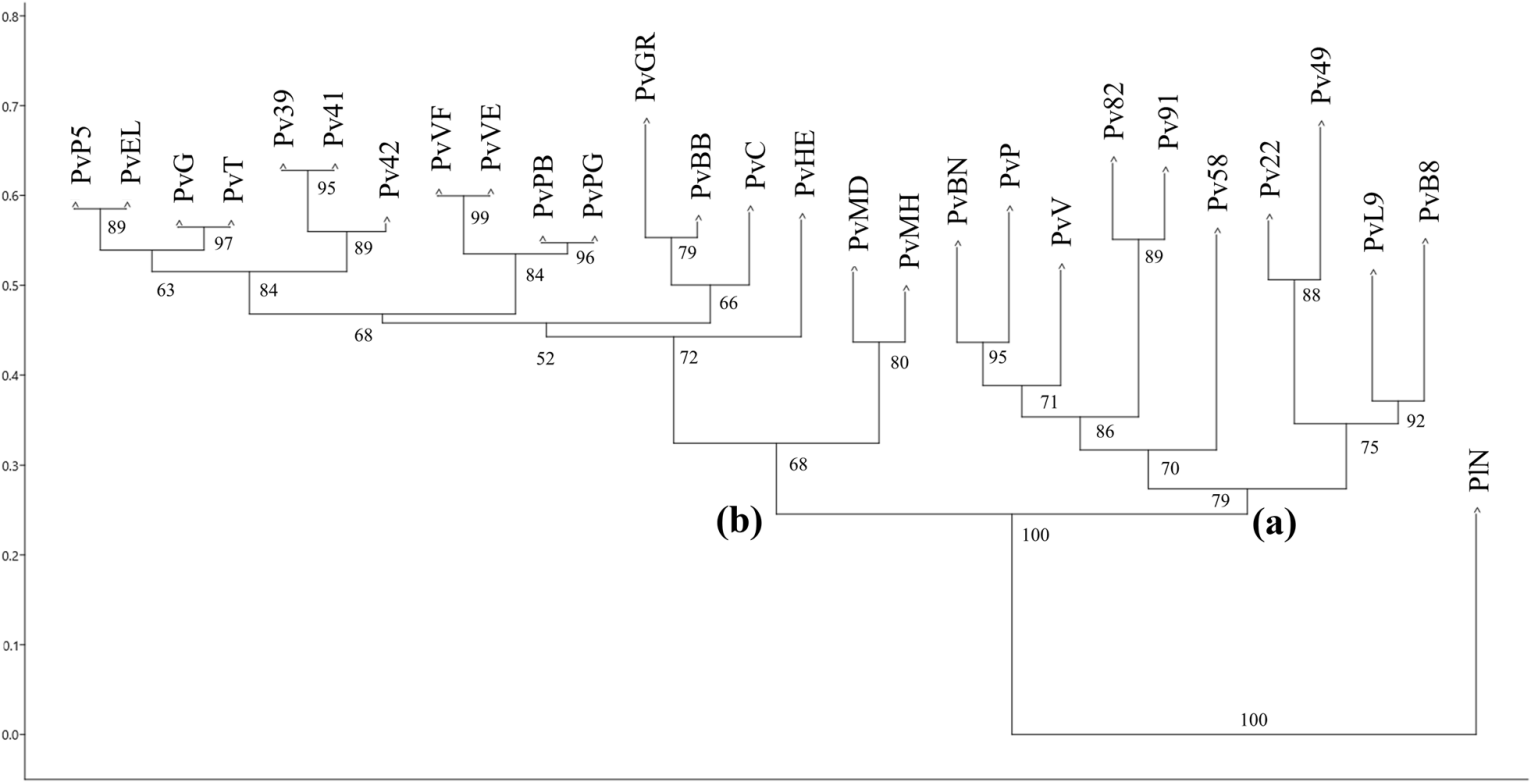
Neighbor joining tree obtained by genetic distances estimated among bean accessions. Two different subgroups can be recognized (**a**) and (**b**). Number at the branch nodes report the bootstrap value probability estimated with 1000 replicates

The cophenetic correlation coefficient was computed resulting in a good measure of the degree of fit (0.952) between data (dissimilarity matrix) and the cluster distribution of the derived tree. The resulting neighbor-joining tree groups the analysed bean accessions in two distantly related clusters, according to the two analysed species *P. lunatus* and *P. vulgaris* (Fig. 7). Within the *P. vulgaris* cluster, two sub-clusters can be recognized. A sub-cluster (a) includes and characterizes accessions with Mesoamerican origin Pv58, Pv22, Pv49, PvL9, PvB8, Pv82, Pv91, with only one exception, an old Italian cultivar ‘Bonello’ (PvBN). In this sub-cluster the two analysed Mexican beans (PvP and PvV) are also included with moderate level of divergence together with PvBN. Despite the very limited information available for this accession, bean characterization studies performed by Lioi L [66] on phaseolin pattern variations within an old word collection of *P. vulgaris* and Mexican wild accessions, reported for the accession PvBN a very rare banding type, clearly different from all the others.

The second major sub-group (b) contains the entire European genetic pool, characterized by lower genetic dissimilarity values when compared to the sub-cluster (a). The sub-group (b) includes all the analysed Italian landraces for which two distinct seed pools were considered, most of them sharing the same TBP profile (ecotypes ‘Poverella’ PB-PG and ‘Verdolino’ VF-VE). In addition, some inner groups can be also recognized within the sub-cluster (b): the bean back-cross lines (Pv39, 41 and 42) group together with the parents involved in their origin, PvT and the recurrent parent PvHE. The Taylor’s Dwarf Horticultural bean (PvT) appears to be identical to an Italian commercial cultivar (PvG) grouping with the third back-cross line of this latter (PvP5).

The Peruvian popping bean (PvEL), the only available Andean accession, clusters with the European gene pool accessions in the sub-cluster (b), corroborating the evidences recognized by several authors [67–69] of the occurrence of two clearly independent domestication events, Mesoamerican and Andean, that originated the European germplasm for bean. In addition, according to studies based on phaseolin and molecular markers, the Andean gene pool of the common bean is always prevalent in the European accessions, accounting for 66% to 76% of the total [68, 70, 71]. Moreover, the genetic characterization performed by SSR molecular markers reported by Lioi et al. [72] documented an Andean origin for the Italian landraces ‘Verdolino’, ‘Ciuoto’ and ‘Bianco di Bagnasco’ (PvVF, PvVE, PvC and PvBB). Further assessed by additional phaseolin pattern variation studies [73], this Italian-Andean germplasm association (sub-cluster (b)) has now been emphasized and confirmed by our TBP cluster analysis.

## Conclusions

The purpose of this contribution was twofold. First, we wanted to provide all the experimental details that make the TBP protocol successfully applicable to the easy genotyping of a vast range of plants, amending possible technical flaws in the information we had released in previous works. The motivation was to provide an additional tool to the scientific community for investigating plant genetic diversity. The second goal was to demonstrate the advantages and the versatility of the TBP method. Chiefly, we demonstrated its capacity to provide consistent genome profiles across different taxonomic ranks from genera to landraces and ecotypes. Accordingly, we have presented data on different genera within papilionoids, different *Arachis* species and subspecies as well as different accessions of *Phaseolus vulgaris*. We hope that this paper will facilitate the use of the TBP method of genotyping by an increasing number of laboratories since, at the very least, it may offer a complementary and more sustainable resource for initial screenings where money and equipment for genomics are not available.

## Supplementary information

**Additional file 1.** CE-TBP data *Arachis* ssp.

**Additional file 2.** CE-TBP data *Phaseolus* L.

## Data Availability

All data generated or analysed during this study are included in this published article.
